# Kriging with Unknown Variance Components for Regional Ionospheric Reconstruction

**DOI:** 10.3390/s17030468

**Published:** 2017-02-27

**Authors:** Ling Huang, Hongping Zhang, Peiliang Xu, Jianghui Geng, Cheng Wang, Jingnan Liu

**Affiliations:** 1 GNSS Research Center, Wuhan University, 129 Luoyu Road, 430079 Wuhan, China; huangling_gnss@whu.edu.cn (L.H.); jgeng@whu.edu.cn (J.G.); acheng@whu.edu.cn (C.W.); jnliu@whu.edu.cn (J.L.); 2 Disaster Prevention Research Institute, Kyoto University, 611-0011 Kyoto, Japan; pxu@rcep.dpri.kyoto-u.ac.jp

**Keywords:** ionospheric delays, Kriging spatial interpolation, semivariogram, variance component estimation, CMONOC, GNSS

## Abstract

Ionospheric delay effect is a critical issue that limits the accuracy of precise Global Navigation Satellite System (GNSS) positioning and navigation for single-frequency users, especially in mid- and low-latitude regions where variations in the ionosphere are larger. Kriging spatial interpolation techniques have been recently introduced to model the spatial correlation and variability of ionosphere, which intrinsically assume that the ionosphere field is stochastically stationary but does not take the random observational errors into account. In this paper, by treating the spatial statistical information on ionosphere as prior knowledge and based on Total Electron Content (TEC) semivariogram analysis, we use Kriging techniques to spatially interpolate TEC values. By assuming that the stochastic models of both the ionospheric signals and measurement errors are only known up to some unknown factors, we propose a new Kriging spatial interpolation method with unknown variance components for both the signals of ionosphere and TEC measurements. Variance component estimation has been integrated with Kriging to reconstruct regional ionospheric delays. The method has been applied to data from the Crustal Movement Observation Network of China (CMONOC) and compared with the ordinary Kriging and polynomial interpolations with spherical cap harmonic functions, polynomial functions and low-degree spherical harmonic functions. The statistics of results indicate that the daily ionospheric variations during the experimental period characterized by the proposed approach have good agreement with the other methods, ranging from 10 to 80 TEC Unit (TECU, 1 TECU = 1 × 10^16^ electrons/m^2^) with an overall mean of 28.2 TECU. The proposed method can produce more appropriate estimations whose general TEC level is as smooth as the ordinary Kriging but with a smaller standard deviation around 3 TECU than others. The residual results show that the interpolation precision of the new proposed method is better than the ordinary Kriging and polynomial interpolation by about 1.2 TECU and 0.7 TECU, respectively. The root mean squared error of the proposed new Kriging with variance components is within 1.5 TECU and is smaller than those from other methods under comparison by about 1 TECU. When compared with ionospheric grid points, the mean squared error of the proposed method is within 6 TECU and smaller than Kriging, indicating that the proposed method can produce more accurate ionospheric delays and better estimation accuracy over China regional area.

## 1. Introduction

The ionosphere is the upper part of atmosphere located between from 50 km to 1300 km above the Earth’s surface. It has a high density of ions and free electrons that can affect the propagation of electromagnetic radio frequency waves [1]. Ionospheric delay is an important source of errors in Global Navigation Satellite System (GNSS) positioning and navigation, which can be determined from Total Electron Content (TEC) measurements. Accurate ionospheric delay corrections can significantly accelerate the convergence of real-time GNSS ambiguity resolution and thus essentially improve precision and performance of positioning and navigation products for single-frequency users [2].

Ionospheric models can be generally classified into two categories: function-based and grid-based [3]. The former represents regional ionospheric TEC by estimating the coefficients of the employed mathematical functions, such as (generalized) trigonometric series functions [4,5], polynomial functions, low-degree spherical harmonic functions and spherical cap harmonic functions [3]. Mathematical function-based models cannot effectively reflect high-frequency variations in local ionosphere, unless the functions employed possess high frequency components. Grid-based ionosphere models have often been adopted by single-frequency users in wide area augmentation systems such as Wide Area Augmentation System (WAAS) and European Geostationary Navigation Overlay Service (EGNOS). Chao (1997) proposed Inverse Distance Weighted (IDW) functions with the Klobuchar model to compute ionospheric delays in WAAS [6]. Komjathy et al. suggested a planar fitting method to interpolate regional ionospheric grid delays [7]. Then WAAS uses a spatial correlation interpolation scheme of Kriging [8], while EGNOS employs a non-uniform partitioning scheme for ionospheric grid [9]. Moreover, grid-based models can be more effective in detail description of local ionosphere variations.

Ionosphere changes spatially and temporally [8]. Kriging (see e.g., [10]), though originating in the field of mining, is developed to fully account for spatio-temporal information on data [10]. Since then, it has become a powerful tool in geostatistics and spatial statistics to handle spatially and/or temporally correlated and irregularly distributed data and has been widely applied to other fields such as hydrology [11], climatology [12], soil science [13], ecology [14], Geo-Information System (GIS) [15], atmosphere science [16], geophysics [17] and geodesy [18]. Since 2002, it has been shown to be efficient for ionospheric delay estimation as well. Blanch conducted extensive experiments to validate the technique of Kriging for ionospheric estimation by using WAAS ionospheric measurements collected during quiet and disturbed periods [8]. Blanch et al. further developed a hybrid algorithm by combining Kriging and tomography for Satellite Based Augmentation Systems (SBAS) and applied it to post-process ionospheric TEC measurements from the US and Brazil [19]. Wielgosz et al. compared Kriging with multiquadratic models by using GPS observations from five Ohio CORS stations; the results have shown that both methods are suitable for instantaneous regional ionosphere modeling [20]. As one of the Internal GNSS Service (IGS) ionosphere analysis centers, Technical University of Catalonia (UPC) has adopted Kriging to re-process existing UPC Global Ionospheric Map (GIM) products. The results have shown that the Root Mean Squared error (RMS) of the UPC Kriging GIM is about 16% lower than the current UPC GIM and about 2% lower than IGS GIM, where the RMS is the root mean square of the difference among the geometry free linear combination observations and the Slant TEC (STEC) computed by each GIM at the same elevation in a continue arch at two different time steps. Both the standard deviation and the RMS are reduced approximately by 0.3 TEC Unit (TECU) (6%) and 0.1 TECU (3%) over the current UPC GIM products when compared with TOPEX/Poseidon and JASON TEC data, respectively [21]. These studies have shown that the Kriging method is useful and effective for ionospheric TEC estimation.

Sayin et al. compared the performance of the Ordinary Kriging (OK) with the Universal Kriging (UK) using a synthetic data set with different variances and different types of sampling patterns. They observed that for small sampling numbers and with higher variability, OK performs better. However, UK gives better results in case of smaller variances in synthetic surfaces and increasing sample number [22]. Although UK takes the random signals of TEC observations into account, it does not consider measurement errors. Li et al. demonstrated, with the Ground-based Regional Integrity Monitoring System (GRIMS) reference stations in China, that UK can produce a more accurate ionospheric delay correction than the distance-weighted method and a tight confidence bound in the boundary areas [23]. The technique has also been well used to reconstruct the ionosphere critical frequency (*f*oF2) instantaneous mapping [24,25,26]. Relevant studies can be found in Tierno et al. [27], Deviren et al. [28] and Chen [29].

Trend and signal in delays reflect the variations in the ionosphere i.e., the TEC [8]. The accuracy of measurements and the stochastic model of signals are two basic elements in Kriging. However, TEC measurement noise has often been neglected in the estimation of ionospheric delay, as can be found in the research works mentioned above. Additionally, in the real conditions, TEC measurements can be of different accuracy. Nevertheless, Kriging cannot be able to handle the situation with a number of different unknown variance components of spatial/temporal data. Therefore, as the first motivation of this paper, we will consider the estimation of ionospheric delays with TEC measurements of different (unknown) accuracy. On the other hand, even if measurement noise and the stochastic model of the signal would be fully taken into account in the estimation of ionospheric delays, we have to determine whether the level of measurement noise and the stochastic model of signals are correctly given, since incorrect stochastic models can distort the estimation of trends. The second motivation of this paper is to apply the new Kriging method to real data and to demonstrate how to eliminate the distortion by calibrating stochastic models of measurements and signals. More specifically, we will theoretically extend the basic Kriging principle to the case with measurements of different (unknown) variance components and calibrate factors for signals to balance the stochastic models of both measurements and signals.

This paper is organized as follows: in Section 2, we will briefly outline ordinary Kriging algorithms and semivariogram for the construction of signal stochastics. In Section 3, we will theoretically extend Kriging methods to the case in which the stochastic model of measurements contains a number of unknown variance components. The general formulae to estimate trend parameters, signals and the unknown stochastic models of measurements will be worked out in detail. The proposed new Kriging method will then be adapted for use in ionospheric TEC estimation in Section 4 and its implementation will be outlined as well. A brief overview of the common used ionospheric mapping models are given in Section 5 in order to compare with our introduced method. In Section 6, we will apply the new method developed in Section 3 and Section 4 to analyze the data from CMONOC and to evaluate the quality of China Regional Ionospheric Maps (CRIM). Finally, the conclusions derived are summed up in Section 7.

## 2. Kriging Spatial Interpolation

### 2.1. The Principle of Kriging Spatial Interpolation

Kriging originates from the field of mining and was developed to deal with spatio-temporally correlated data [30]. Since then, it has become a standard and powerful method in geostatistics and spatial statistics [10] and has found a variety of applications in all the subjects of study where (regularly and/or irregularly) spatially and/or temporally correlated data are routinely encountered. It is based on the variability and spatial correlation of regionalized variables to determine the weights of sampling points distributed around the point to be estimated, according to the principle of unbiased and optimal estimation. Finally, the value of the estimated point is obtained by using the linear combination of data samples. The spatial variability and correlation of data is described by using spatial covariance function or semivariogram. Very often, we do not know the semivariogram in advance. Instead, it must be practically estimated from the original data set. In the remainder of this section, we will closely follow Cressie to briefly outline the basic principle of the ordinary Kriging.

In geostatistics, we usually assume the stationarity for spatially distributed data. To be specific, given a spatial random function *Z*(**x**), we assume (i) that its expectation is constant and does not depend on the location **x** and (ii) that the correlation function between any two points depends solely on their distance. These two assumptions can be mathematically described equivalently as follows:

(1) $$\begin{array}{l} E\left[Z\left(x\right)\right]=m,\;\forall x\\ E\left[Z\left(x+h\right)-Z\left(x\right)\right]=0 \end{array}$$

(2) $$\begin{array}{ll} Var\left[Z\left(x+h\right)-Z\left(x\right)\right]= & E\left\{{\left\{\left[Z\left(x+h\right)-Z\left(x\right)\right]-E\left[Z\left(x+h\right)-Z\left(x\right)\right]\right\}}^{2}\right\}\\ & =E\left\{{\left[Z\left(x+h\right)-Z\left(x\right)\right]}^{2}\right\}=2\gamma \left(h\right) \end{array}$$

(see e.g., Cressie [23]), where the function γ(h) is called semivariogram, which characterizes the spatial correlation of random function Z(x), and h=‖h‖ is the distance between the two spatial points. If the assumption of isotropy is removed, then *h* will not be a scalar of distance but should be replaced by the vector between the two points. If we further remove the assumption of homogeneity, in this case, the semivariogram should be written in its most general form as γ(x+h,x).

Based on the above assumptions and given a semivariogram function, the basic Kriging method is to find the best linear unbiased estimator or interpolator with minimum variance. Given a set of measurements $Z\left(x_{1}\right)$, $Z\left(x_{2}\right)$, ...,$Z\left(x_{N}\right)$, we can interpolate the value $\hat{Z}\left(x_{0}\right)$ of a given (non-measured) point *x*_0_ by constructing a linear combination of the measurements as follows:

(3) $$\hat{Z}\left(x_{0}\right)=\sum_{i=1}^{N}\lambda_{i}Z\left(x_{i}\right)$$

where $\lambda_{i}$ are the unknown coefficients to be determined. In general, we also require that these coefficients be non-negative.

The expectation of the difference between the linear interpolation Equation (3) and the signal at the point $x_{0}$ can be written as follows:

(4) $$E\left(\hat{Z}\left(x_{0}\right)-Z\left(x_{0}\right)\right)=E\left(\sum_{i=1}^{N}\lambda_{i}Z\left(x_{i}\right)-Z\left(x_{0}\right)\right)=\sum_{i=1}^{N}\lambda_{i}E\left(Z\left(x_{i}\right)\right)-E\left(Z\left(x_{0}\right)\right)=m\;\left(\sum_{i=1}^{N}\lambda_{i}-1\right)$$

Since we require the linear interpolator Equation (3) be unbiased, and bearing the condition Equation (1) in mind, we must have:

(5) $$\sum_{i=1}^{N}\lambda_{i}=1$$

Under condition Equation (5), we can further compute the error variance for $\hat{Z}\left(x_{0}\right)-Z\left(x_{0}\right)$, which is given below:

(6) $$\mathrm{var}\left(\hat{Z}\left(x_{0}\right)-Z\left(x_{0}\right)\right)=2\sum_{i=1}^{N}\lambda_{i}\gamma \left(x_{i},x_{0}\right)-\sum_{i=1}^{N}\sum_{j=1}^{N}\lambda_{i}\lambda_{j}\gamma \left(x_{i},x_{j}\right)-\gamma \left(x_{0},x_{0}\right)$$

where $\gamma \left(x_{i},x_{j}\right)$ is the semivariogram between points $x_{i}$ and $x_{j}$.

To construct the optimal interpolator Equation (3), we require minimum error variance Equation (6) under the constraint Equation (5) of unbiasedness. Following Cressie [23], we construct the augmented objective function:

(7) $$L\left(\lambda ,\mu \right)=2\sum_{i=1}^{N}\lambda_{i}\gamma \left(x_{i},x_{0}\right)-\sum_{i=1}^{N}\sum_{j=1}^{N}\lambda_{i}\lambda_{j}\gamma \left(x_{i},x_{j}\right)-\gamma \left(x_{0},x_{0}\right)-2\mu \left(\sum_{i=0}^{N}\lambda_{i}-1\right)$$

where μ is the Lagrange multiplier. By computing the partial derivatives of the objective function Equation (7) with respect to $\lambda_{i}$ and setting them to zero, we have the normal equations:

(8) $$\sum_{i=1}^{N}\lambda_{i}\gamma \left(x_{i},x_{j}\right)+\mu =\gamma \left(x_{j},x_{0}\right)\;,\;j=1,\;2,\cdots ,N$$

from which, together with the equality condition Equation (5) of unbiasedness, we can readily obtain the following linear system of equations:

(9) $$\left[\begin{array}{lllll} \gamma_{11} & \gamma_{12} & \cdots & \gamma_{1N} & 1\\ \gamma_{21} & \gamma_{22} & \cdots & \gamma_{2N} & 1\\ \;\vdots & \vdots & \ddots & \;\vdots & \vdots \\ \gamma_{N1} & \gamma_{N2} & \cdots & \gamma_{NN} & 1\\ \;1 & 1 & \cdots & 1 & 0 \end{array}\right]\left[\begin{array}{c} \lambda_{1}\\ \lambda_{2}\\ \;\vdots \\ \lambda_{N}\\ \mu \end{array}\right]=\left[\begin{array}{l} \gamma_{10}\\ \gamma_{20}\\ \;\vdots \\ \gamma_{N0}\\ \;1 \end{array}\right]$$

By solving the Equation (9), we have the solution of $\lambda_{i}$ and μ, and as a result, can use them to construct the OK interpolator Equation (3) and to complete the estimation of $\hat{Z}\left(x_{0}\right)$. Here $\gamma_{ij}$ (*i*, *j* = 1, 2 ,..., *N*) stands for $\gamma \left(x_{i},x_{j}\right)$ in Equation (8), and $\gamma_{i0}$ stands for the semivariogram between the *i*-th measured point and the point to be interpolated, for the conciseness of notations. The estimated error variance of the optimal OK interpolator can be derived by applying the error propagation law to Equation (3) and is simply given as follows:

(10) $${\hat{\sigma }}_{OK}^{2}=\sum_{i=1}^{N}\lambda_{i}\gamma \left(x_{0},x_{i}\right)-\gamma \left(x_{0},x_{0}\right)+\mu$$

Equations (9) and (10) can also be written alternatively in matrix form as follows:

(11) Βλ=Γ

(12) $${\hat{\sigma }}_{OK}^{2}=\Gamma^{T}B^{-1}\Gamma -\Gamma_{0}$$

where:

(13) $$B=\left[\begin{array}{lllll} \gamma_{11} & \gamma_{12} & \cdots & \gamma_{1N} & 1\\ \gamma_{21} & \gamma_{22} & \cdots & \gamma_{2N} & 1\\ \;\vdots & \;\vdots & \ddots & \;\vdots & \vdots \\ \gamma_{N1} & \gamma_{N2} & \cdots & \gamma_{NN} & 1\\ \;1 & 1 & \cdots & 1 & 0 \end{array}\right]$$

(14) $$\Gamma ={\left[\gamma_{10}\;\gamma_{20}\;\cdots \;\gamma_{N0}\;1\right]}^{T},\;\lambda ={\left[\lambda_{1}\;\lambda_{2}\;\cdots \;\lambda_{N}\;\mu \right]}^{T},\;\Gamma_{0}=\left[\gamma_{00}\right]$$

Therefore, the weights $\lambda_{i}$ and the Lagrange multiplier μ can be estimated by solving Equation (11).

### 2.2. Construction of Semivariogram

A semivariogram describes the spatial correlation of a random field and plays a key role in spatial Kriging interpolation. In practice, a semivariogram is generally unknown but has to be estimated from spatial measurements. To simplify the numerical representation of spatial correlations among random points under the assumption of stationarity and isotropy, the semivariogram in this case is often represented by using a few parameters [30].

By definition, a semivariogram is half of the variance of the difference between Z(x) and Z(x+h), which is related to the covariance function through the following relationship [30]:

(15) $$C\left(x_{k},x_{l}\right)=\sigma_{\infty }^{2}-\gamma \left(x_{k},x_{l}\right)=C\left(0\right)-\gamma \left(x_{k},x_{l}\right)$$

Given *N*(*h*) pairs of measurements separated at a distance *h* and under the assumption of stationarity and isotropy, the semivariogram can then be numerically estimated as follows:

(16) $$\gamma \left(h\right)=\frac{1}{2N\left(h\right)}\sum_{\left[h-\delta ,h+\delta \right]}^{N\left(h\right)}{\left(Z\left(x_{k}\right)-Z\left(x_{l}\right)\right)}^{2}$$

Practically, all the measurement points may not be regularly distributed in space. In this case, we will have to allow a certain tolerance in order to have a sufficient number of data pairs to estimate semivariogram. Nevertheless, a large number of semivariogram values, as given by Equation (16), may not be convenient to use. Thus, one often selects some appropriate and simplified models to fit and represent semivariogram. The most common used semivariogram models include spherical functions, exponential functions and Gaussian functions [8,30].

## 3. Variance Component Estimation (VCE) Based on Collocation

Variance component estimation (VCE) has been one of the most important topics in geodesy [31,32,33,34,35]. To correctly determine the weights of different types of measurements and/or measurements of different precisions, we have to simultaneously estimate both the model parameters and variance components. A number of methods have been proposed both in geodesy and statistics to estimate the unknown parameters along with the unknown stochastic model of measurements. The most widely used methods include Helmert quadratic estimation [32], maximum likelihood [33], the Best Invariant Quadratic Unbiased Estimation (BIQUE) [34], the Restricted Maximum Likelihood Method (REML) [36], and the MInimum Norm Quadratic Unbiased Estimation (MINQUE) [37,38]. Extensions to ill-posed problems can be found in Xu et al. [35], Xu [39] and Eshagh [40].

In this section, we focus on the following collocation model:

(17) y=Aβ+Bs+ε

where **y** is an *n* × 1 observation vector. In the literature of ionospheric modeling, the notation **I**(**x**) is often used instead of **y**, to denote observations of ionospheric delays. ε is the corresponding measurement error vector; **A** is the (*n* × *t*) design matrix of the unknown parameters, β is a *t* × 1 vector that contains (deterministic) unknown trend parameters to be estimated; **s** is an *m* × 1 vector of random signals with an associated (*n* × *m*) design matrix **B**.

In general, the least-squares collocation method usually assumes that the variance-covariance matrices of the measurement errors and the signals are given and then uses the measurements **y** to estimate the parameters β and the random signals s. Yang and Xu extended the collocation model to the case that allows one unknown variance component for the measurements **y** and one unknown variance component for the random signals **s** [41]. Yang et al. introduced an adaptive factor into a new adaptive collocation procedure and used the maximum likelihood technique to determine the weights of the signals and measurements [42].

In this paper, we will further extend the collocation model to a very general case with a number of unknown variance components for the stochastic models of both the measurements ε and the random signals **s**. For simplicity, we will focus on the following stochastic model:

(18) $$\begin{array}{l} E\left(s\right)=0,\;E\left(\varepsilon \right)=0\\ D\left[\begin{array}{l} s\\ \varepsilon \end{array}\right]=\Sigma =\left[\begin{array}{cc} \Sigma_{s} & 0\\ 0 & \Sigma_{\varepsilon } \end{array}\right]=\left[\begin{array}{cc} \sum_{i=1}^{k_{S}}U_{si}\sigma_{si}^{2} & 0\\ 0 & \sum_{i=1}^{k_{\varepsilon }}U_{\varepsilon i}\sigma_{\varepsilon i}^{2} \end{array}\right] \end{array}$$

where the variance-covariance matrix Σ contains $\Sigma_{\varepsilon }$ and $\Sigma_{s}$ that are the variance-covariance matrices of the measurements and the random signals, respectively. $U_{si}$ and $U_{\varepsilon i}$ are the given positive (semi-) definite matrices, and $\sigma_{si}^{2}$ (*i* = 1,2,…,$k_{s}$) and $\sigma_{\varepsilon i}^{2}$ (*i* = 1,2,…,$k_{\varepsilon }$) are the unknown variance components. If the measurements and the random signals can be divided into a number of stochastically independent sub-groups, and if the measurements and the random signals are stochastically independent as well, then $\Sigma_{s}$ and $\Sigma_{\varepsilon }$ become block-diagonal, which can be rewritten as follows:

(19) $$\Sigma_{s}=\left[\begin{array}{cccc} P_{s1}^{-1}\sigma_{s1}^{2} & 0 & \cdots & 0\\ 0 & P_{s2}^{-1}\sigma_{s2}^{2} & \cdots & 0\\ \vdots & \vdots & \ddots & \vdots \\ 0 & 0 & \cdots & P_{sk_{s}}^{-1}\sigma_{sk_{s}}^{2} \end{array}\right],\Sigma_{\varepsilon }=\left[\begin{array}{cccc} P_{\varepsilon 1}^{-1}\sigma_{\varepsilon 1}^{2} & 0 & \cdots & 0\\ 0 & P_{\varepsilon 2}^{-1}\sigma_{\varepsilon 2}^{2} & \cdots & 0\\ \vdots & \vdots & \ddots & \vdots \\ 0 & 0 & \cdots & P_{\varepsilon k_{\varepsilon }}^{-1}\sigma_{\varepsilon k_{\varepsilon }}^{2} \end{array}\right]$$

where $P_{si}$ and $P_{\varepsilon i}$ are the weight matrices, corresponding, respectively, to the *i*-th sub-group of the random signals **s** and the measurement errors ε. In this case, the matrices $U_{si}$ and $U_{\varepsilon i}$ become:

(20) $$U_{si}=\left[\begin{array}{cccc} 0 & 0 & \cdots & 0\\ 0 & P_{si}^{-1} & \cdots & 0\\ \vdots & \vdots & \ddots & \vdots \\ 0 & 0 & 0 & 0 \end{array}\right],\;i=1,2,\ldots ,k_{s}$$

and:

(21) $$U_{\varepsilon i}=\left[\begin{array}{cccc} 0 & 0 & \cdots & 0\\ 0 & P_{\varepsilon i}^{-1} & \cdots & 0\\ \vdots & \vdots & \ddots & \vdots \\ 0 & 0 & 0 & 0 \end{array}\right],\;i=1,2,\ldots ,k_{\varepsilon }$$

If the signal vector $s^{\prime }$ at unmeasured points is also included into the collocation model (16), we then have:

(22) $$y=A\beta +\left[B\;0\right]\;\left[\begin{array}{l} s\\ s^{\prime } \end{array}\right]+\varepsilon$$

If the variance components in the stochastic models is known or given with some initial values, we apply the least squares collocation principle (see e.g., Huang 1992) [43] to model (22) and obtain the following solution:

(23) $$\begin{array}{l} \hat{\beta }={\left(A^{T}P_{y}A\right)}^{-1}A^{T}P_{y}y\\ \hat{s}=\Sigma_{s}B^{T}P_{y}\left(y-A\hat{\beta }\right)\\ {\hat{s}}^{\prime }=\Sigma_{s^{\prime }s}B^{T}P_{y}\left(y-A\hat{\beta }\right) \end{array}$$

where $P_{y}={\left(B\Sigma_{s}B^{T}+\Sigma_{\varepsilon }\right)}^{-1}$, $\hat{\beta }$ is the estimated trend parameter vector, $\hat{s}$ is the estimated signal vector at the known points, ${\hat{s}}^{\prime }$ is the estimated signal vector at the unmeasured points.

Since the variance components are unknown, we now apply variance component estimation to the collocation model (22) to estimate the variance components of the measurements and the random signals. Specifically, we will use the MINQUE method in this section.

To estimate the variance components for the measurements and the random signals, and keeping in mind that the variance component estimation has nothing to do with the unobserved signals, we rewrite the stochastic signals as pseudo observations. Thus, the collocation model (17) can be alternatively presented as follows:

(24) $$\begin{array}{l} y=A\beta +Bs+\varepsilon ,\;\Sigma_{\varepsilon }=\sum_{i=1}^{k_{\varepsilon }}U_{\varepsilon i}\sigma_{\varepsilon i}^{2}\\ y_{s}=s+\varepsilon_{s},\;\Sigma_{s}=\sum_{i=1}^{k_{s}}U_{si}\sigma_{si}^{2} \end{array}\}$$

where $y_{s}$ and $\varepsilon_{s}$ denotes the prior values and errors of the signals, respectively.

Given some initial values $\sigma_{s0}^{2}={\left[\sigma_{s10}^{2}\;\sigma_{s20}^{2}\cdots \sigma_{sk_{s}0}^{2}\right]}^{T}$ and $\sigma_{\varepsilon 0}^{2}={\left[\sigma_{\varepsilon 10}^{2}\;\sigma_{\varepsilon 20}^{2}\cdots \sigma_{\varepsilon k_{\varepsilon }0}^{2}\right]}^{T}$ for the unknown variance components, the MINQUE estimate of the variance components can be computed by using the following equations (see e.g., [35,37,43]):

(25) $$G\hat{\sigma }=q$$

where:

(26) $$G=\left[\begin{array}{ll} G_{\varepsilon } & G_{\varepsilon s}\\ G_{s\varepsilon } & G_{ss} \end{array}\right]$$

(27) $$\hat{\sigma }=\left[\begin{array}{l} {\hat{\sigma }}_{\varepsilon }^{2}\\ {\hat{\sigma }}_{s}^{2} \end{array}\right]$$

(28) $$q=\left[\begin{array}{l} q_{\varepsilon }\\ q_{s} \end{array}\right]$$

and the elements of the coefficient matrix **G** and vector **q** are given by:

(29) $$\begin{array}{ll} g_{\varepsilon }^{ij}=tr\left(CU_{\varepsilon }^{i}CU_{\varepsilon }^{j}\right)\;, & \left(i,j=1,2,\ldots ,k_{\varepsilon }\right)\\ g_{\varepsilon s}^{ij}=tr\left(CU_{\varepsilon }^{i}CU_{s}^{j}\right)\;, & \left(i=1,2,\ldots ,k_{\varepsilon };\;j=1,2,\ldots ,k_{s}\right)\\ g_{s\varepsilon }^{ij}=tr\left(CU_{s}^{i}CU_{\varepsilon }^{j}\right)\;, & \left(i=1,2,\ldots ,k_{s};\;j=1,2,\ldots ,k_{\varepsilon }\right)\\ g_{s}^{ij}=tr\left(CU_{s}^{i}CU_{s}^{j}\right)\;, & \left(i,j=1,2,\ldots ,k_{s}\right) \end{array}$$

(30) $$q_{\varepsilon }^{i}=V^{T}CU_{\varepsilon }^{i}CV\;,\;\left(i=1,2,\cdots ,k_{\varepsilon }\right)$$

(31) $$q_{s}^{i}=V^{T}CU_{s}^{i}CV\;,\;\left(i=1,2,\cdots ,k_{s}\right)$$

*tr*(·) denotes the trace of a square matrix, and:

(32) $$F=\left[\begin{array}{ll} A & B\\ 0 & I \end{array}\right]$$

(33) $$C=\Sigma_{0}^{-1}-\Sigma_{0}^{-1}F{\left(F^{T}\Sigma_{0}^{-1}F\right)}^{-1}F^{T}\Sigma_{0}^{-1}$$

(34) $$U_{\varepsilon }^{i}=\left[\begin{array}{cc} U_{\varepsilon i} & 0\\ 0 & 0 \end{array}\right],\;\left(i=1,2,\cdots ,k_{\varepsilon }\right),\;U_{s}^{i}=\left[\begin{array}{cc} 0 & 0\\ 0 & U_{si} \end{array}\right],\;\left(i=1,2,\cdots ,k_{s}\right)$$

(35) $$V=\left[\begin{array}{l} V_{\varepsilon }\\ V_{s} \end{array}\right],\;V_{\varepsilon }=A{\hat{\beta }}_{0}+B{\hat{s}}_{0}-y,\;V_{s}={\hat{s}}_{0}$$

$\Sigma_{0}$ stands for Σ with initial sets of $\sigma_{s0}^{2}$ and $\sigma_{\varepsilon 0}^{2}$. After obtaining the estimates of ${\hat{\sigma }}_{s0}^{2}$ and ${\hat{\sigma }}_{\varepsilon 0}^{2}$, we can re-estimate $\hat{\beta }$, $\hat{s}$ and ${\hat{s}}^{\prime }$ in Equation (23) and further use these Equations (22)–(35) to iteratively estimate the variance components. Then the estimations ${\hat{y}}^{\prime }$ at unmeasured points can be get by the following Equation (36):

(36) $$\hat{y^{\prime }}=A^{\prime }\hat{\beta }+B^{\prime }{\hat{s}}^{\prime }$$

and the corresponding variance-covariance matrix $\Sigma_{\hat{y^{\prime }}}$ is:

(37) $$\Sigma_{\hat{y^{\prime }}}=\left[\begin{array}{cc} A^{\prime } & B^{\prime } \end{array}\right]\left[\begin{array}{cc} \Sigma_{\hat{\beta }} & \Sigma_{\hat{\beta }{\hat{s}}^{\prime }}\\ \Sigma_{{\hat{s}}^{\prime }\hat{\beta }} & \Sigma_{\hat{\beta }} \end{array}\right]{\left[\begin{array}{c} A^{\prime }\\ B^{\prime } \end{array}\right]}^{T}$$

where $A^{\prime }$ and $B^{\prime }$ are the design matrices, and the formulas of variance matrices in Equation (37) can refer to Huang [43] in detail.

## 4. Implementation of VCE Integrating with Kriging in Ionospheric Delay Estimation

The observable of STEC can be generated by forming the geometry-free linear combination of pseudorange and phase data. The Vertical TEC (VTEC) can then be derived from STEC at the corresponding ionospheric Intersecting Pierce Point (IPP) by multiplying with the mapping function [44]. In this paper, the Modified Single-Layer Model (MSLM) mapping function is employed [44]. The variance of the TEC observations at different elevations is calculated using the following formula:

(38) $$\Sigma_{\varepsilon i}=\left\{ \begin{array}{ll} 2\sigma_{0}^{2} & ,\;e>30^{{}^{\circ}}\\ \sigma_{0}^{2}/\left(2\sin^{2}\left(e\right)\right) & ,\;e\leq 30^{{}^{\circ}} \end{array}\right.$$

where *e* is the elevation angle of the satellite (the cut-off elevation angle is set to 15°, $\Sigma_{\varepsilon i}$ is the corresponding variance of the ionospheric delay observation error; $\sigma_{0}^{2}$ is the prior variance of the the TEC estimation noise.

For ionospheric delay estimation, we set the trend in the Equation (17) to be constant. Moreover, the matrix **B** is an identity matrix. The observation equation for the vertical ionospheric delay at the IPP located at **x** with **x** ∈ ***φ***, ***λ***, denoted by **I**(**x**), becomes:

(39) I(x)=β+s+ε

For the sake of simplicity in the ordinary Kriging interpolation, we directly use the VTEC observed at IPPs within a certain limit around the Ionospheric Grid Points (IGPs) to interpolate the VTEC according to the algorithm in Section 2.1.

**Figure 1:**
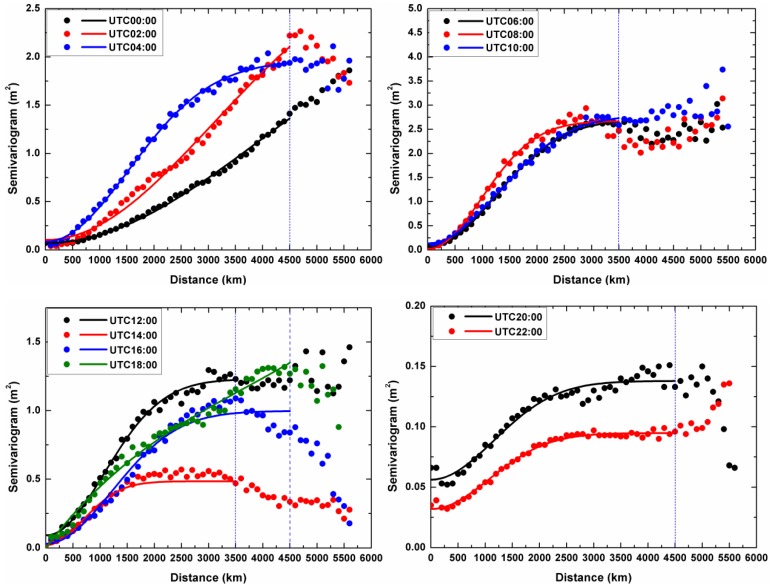
Experimental semivariogram (dots) and the fitting results using Gaussian function (line) at different UTC time on DOY 305, 2014.

**Figure 2:**
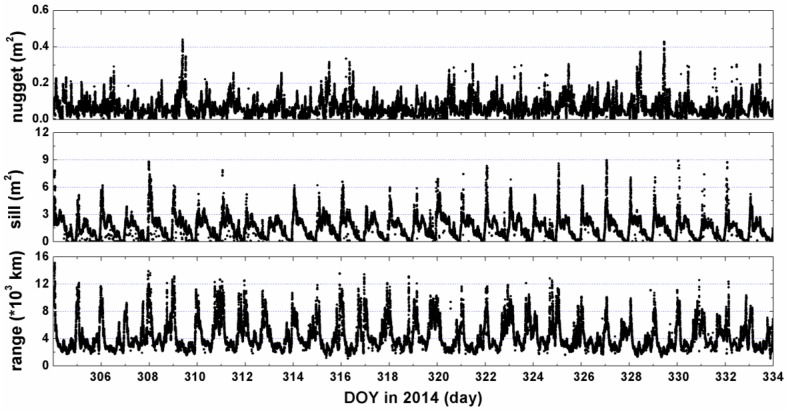
The semivariogram parameters (*nugget*, *sill*, *range*) during 2014.11.

In order to apply Kriging interpolation and VCE for ionospheric estimation, we need to choose some appropriate semivariogram or variance function which can give exact veracious description for ionosphere random behavior. Figure 1 presents the semivariograms of the ionosphere on day-of-year (DOY) 305 in 2014 (2014.11.01) from UTC 00:00 to 22:00 with a 2-h interval which are computed by using Equation (16) based upon CMONOC observational data sets. The dots and curves shown in Figure 1 are the experimental semivariograms and fitting results respectively. According to the shape and behavior of the experimental semivariogram, the Gaussian semivariogram function of the signals is selected:

(40) $$\gamma \left(h\right)=C_{0}+C\left(1-e^{-\frac{h^{2}}{a^{2}}}\right)$$

(see e.g., [30]), where *C*_0_, *C* and *a* are the unknown parameters to be estimated. In geostatistics, *C*_0_ is called “*nugget*” which is linked to the continuity and to the spatial regularity of the regionalized variables. The physical explanations in ionosphere study, the discontinuity at the origin are expounded due to the obliquity error, remaining bias in the measurements and the fast-changing ionosphere [8]. The semivariogram reaches a limiting value, C_0_ ± C, termed as “*sill*” on behalf of the structural variance of spatial variation, and reflect the biggest variation extent of the variables. The two observations are uncorrelated if the distance is beyond a limit. This distance is called the “*range*”. This value is equal to $\sqrt{3}a$ for Gaussian function model [30]. These parameters are estimated by using the data in the corresponding interval of time to characterize the spatial correlation and variability. Therefore, for each epoch map, a set of new semivariogram parameters (*nugget*, *sill*, *range*) is obtained which give descriptions for the behavior of the ionospheric TEC. Figure 2 indicates how the semivariogram changes during the campaign days.

The prior variance-covariance of the signals can be computed according to Equation (15) after fitting the experimental semivariogram to the theoretical semivariogram model (40) by using the least squares estimation. Once the semivariogram and variance are computed, with the known and typically diagonal measurement noise matrix, the Kriging and VCE methods can be used to estimate the results and the estimation variance. Using the developed algorithms, the maps for regional TEC in China can be obtained in real-time with the fitted semivariogram function automatically at any desired epoch.

In order to estimate the VTEC at a given IGP at a given epoch, we must first select a set of VTEC measurements whose IPPs are distributed within the restricted scope centered upon this IGP. We set the maximum radius and minimum radius as $R_{\max}$ = 2000 km and $R_{\min}$ = 500 km respectively. It is important to note that the appropriate radius $\tilde{R}$ for searching IPPs at a given IGP must be within a half of the given searching limits and half of the *range*, as well as the $R_{\max}$. The minimum and maximum number of IPP measurements is set as $N_{\min}$ = 5 and $N_{\max}$ = 25 based on our experiments. If there are IPPs fewer than $N_{\min}$ within $\tilde{R}$, no corresponding estimate is computed. If we find the $N_{\max}$ IPPs around the estimated IGP, then we stop searching. On the other hand, due to the variation level of ionospheric activity which calls for a change for the degree of the ionosphere measurements correlation, the experimental semivariograms have to be fitted in real-time with the IPPs located within appropriate searching limits, with the distance interval to compute the semivariogram from data and its tolerance set to 100 km and 50 km, respectively. The searching limits here refer to the maximum distance between any two IPPs with correlation. From Figure 1, we can see that in the disturbed ionospheric condition, the semivariograms have smaller *range* and larger *sill* than those in the quite ionospheric condition. In the measured data tests, this value of searching limits is set as 3500 km between 14:00 and 22:00 in China local time (=UTC + 8 h), and 4500 km at other moments, as shown in blue vertical dashed lines in Figure 1.

## 5. Regional Ionospheric Models

For the purpose of numerical comparisons in this paper, we will briefly outline some ionospheric interpolation methods used for ionospheric mapping, namely, polynomial interpolations and interpolations with low-degree spherical harmonic functions and spherical cap harmonic analysis.

The polynomial ionospheric interpolation method uses a polynomial function of the latitude differentials and the hour angles differentials of the Sun to interpolate ionospheric delays. The mathematical model can be written as follows:

(41) $$I_{v}\left(\varphi ,\lambda \right)=\sum_{i=0}^{n}\sum_{k=0}^{m}E_{ik}{\left(\varphi -\varphi_{0}\right)}^{i}{\left(S-S_{0}\right)}^{k}$$

(see e.g., [44,45]), where *I_v_* stands for an VTEC measurement, (φ,λ) are the geographic latitude and longitude of the IPP, *E_ik_* are the coefficients of the polynomial function, *S*_0_ is the hour angle of the Sun observed at the central point of the central epoch *t*_0_ in the observation session, e.g., $S-S_{0}=\left(\lambda -\lambda_{0}\right)+\left(t-t_{0}\right)$, $\left(\varphi_{0},\lambda_{0}\right)$ are the central point coordinates of the IPPs, and *t* is the observation epoch. In this study, the degrees (*n*, *m*) of the polynomial function (41) are set to *n* = *m* = 8 for regional modeling and *n* = *m* = 1 for local interpolation, respectively.

The functional method with low-degree spherical harmonic functions is to represent an ionospheric delay measurement with the latitude-dependent associated Legendre functions and the sum of the longitude-dependent sine and cosine terms. The mathematical expression of a VTEC measurement *I_v_* at an IPP with low-degree spherical harmonic functions is given by [3,44]:

(42) $$I_{v}\left(\varphi ,s\right)=\sum_{n=0}^{n_{\max}}\sum_{m=0}^{n}{\tilde{P}}_{nm}\left(\sin\varphi \right)\left({\tilde{C}}_{nm}\cos\left(ms\right)+{\tilde{S}}_{nm}\sin\left(ms\right)\right)$$

where φ is the geographic latitude of the IPP, $s=\lambda -\lambda_{0}$ is the sun-fixed longitude of the IPP, λ is the longitude of the IPP, $\lambda_{0}$ is the longitude of the Sun, *n_max_* is the maximum degree of the spherical harmonic expansion, ${\tilde{P}}_{nm}$ is the normalized associated Legendre function of degree *n* and order *m*, and ${\tilde{C}}_{nm}$ and ${\tilde{S}}_{nm}$ are the spherical harmonic coefficients to be estimated. The degree and order define the resolution of the model, and should be coordinated with the scope of region and the number of measurements [44]. In this paper, the maximum degree is set to 5 experimentally and the total number of the unknown coefficients is ${\left(n_{\max}+1\right)}^{2}=36$.

The regional model on the basis on spherical cap harmonic analysis has also been routinely used in ionospheric modeling [3]. The model with a spherical cap in interval [0, $\theta_{0}$] for regional mapping of VTEC is expressed as:

(43) $$I_{v}\left(\varphi_{c},\lambda_{c}\right)=\sum_{k=0}^{K_{\max}}\sum_{m=0}^{\min\left(k,M\right)}\;{\tilde{P}}_{n_{k}\left(m\right)}\left(\cos\theta_{c}\right)\left({\tilde{C}}_{km}\cos\left(m\lambda_{c}\right)+{\tilde{S}}_{km}\sin\left(m\lambda_{c}\right)\right)$$

where $I_{v}\left(\varphi_{c},\lambda_{c}\right)$ is the VTEC measurement at IPP $\left(\varphi_{c},\lambda_{c}\right)$ inside the spherical cap, *K*_max_ , *M* and *n_k_*_(*m*)_ are the maximum degree, order of the series and the non-integral degrees, respectively. *k* is the index of the non-integral degrees *n_k_*_(*m*)_ (0 ≤ *k* ≤ *K_max_*) computed by an iterative bisection solution. $\tilde{P}\left(\cos\theta_{c}\right)$ is the normalized associated Legendre function, ${\tilde{C}}_{km}$ and ${\tilde{S}}_{km}$ are the unknown normalized spherical cap harmonic coefficients. If the geographic coordinate of the pole of the spherical cap is $\left(\theta_{N},\lambda_{N}\right)$, a point with the geographic coordinates (φ,λ) can be transformed into the spherical cap coordinates as follows (see e.g., [3]):

(44) $$\left\{ \begin{array}{l} \theta_{c}=\arccos\left[\cos\theta_{N}\cos\theta +\sin\theta_{N}\sin\theta \cos\left(\lambda_{N}-\lambda \right)\right]\\ \lambda_{c}=\arcsin\left[\frac{\sin\left(\lambda -\lambda_{N}\right)}{\sin\theta_{c}}\sin\theta \right] \end{array}\right.$$

where the co-latitudes $\theta =90^{\circ }-\varphi$, and $\theta_{c}=90^{\circ }-\varphi_{c}$. The modeling area ranges from 5° to 60° in latitude and from 70° to 140° in longitude, so the pole of the spherical cap $\left(\theta_{N},\lambda_{N}\right)$ and the half angle $\theta_{0}$ are determined as (35°, 105°) and 30°, respectively. In the next section of applications, *K_max_* and *M* are respectively set to 8 and 6 according to Equation (45) to reach the tradeoff between the measurement resolution $\omega_{\min}$ and computational load, and the total number of the unknown coefficients is ${\left(K_{\max}+1\right)}^{2}-(K_{\max}-M)\cdot (K_{\max}-M+1)=75$. More mathematical information on spherical cap harmonic function and the *n_k_*_(*m*)_ solution can be found in Liu [3] and the references therein:

(45) $$K_{\max}\approx \frac{2\theta_{0}}{\pi }\left(\frac{2\pi }{\omega_{\min}}+0.5\right)-0.5$$

## 6. Applications to CMONOC Data and Result Analysis

We have applied Kriging with unknown variance components to reconstruct the ionospheric maps over China, which will be abbreviated as KVCE. We will also compare different methods for ionospheric reconstruction. The local POLYnomial Interpolation (named as IPOLY) approach is also employed. The regional function-based models used include the low-degree SPHerical harmonic function model (SPH), POLYnomial function model (POLY) and Sphere Cap Harmonic Analysis model (SCHA).

**Figure 3:**
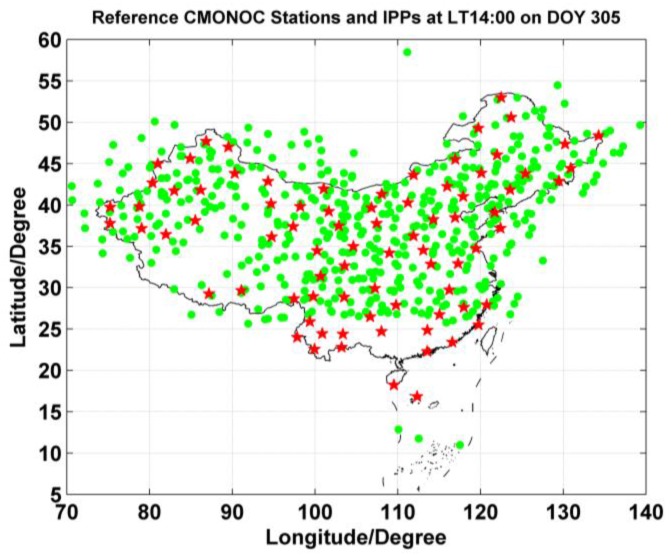
The distribution of the selected reference stations (red pentagram points) and IPPs (green points) at local time 14:00 on DOY 305 of 2014.

The Crustal Movement Observation Network of China (CMONOC) consists of about 260 GPS stations. To demonstrate the construction of a regional real-time ionospheric model over China, 80 stations with a reasonable level of a uniform distribution from CMONOC are selected with data in November 2014 (from DOY 305 to 334). The processing interval is 60s with a cutoff angle of 15° due to high level of errors in low elevation angles. The regional range is (5°–60° N, 70°–140° E) with resolution of 2.5° in latitude and 5° in longitude with a total of 345 grids. Figure 3 shows the distributions of the selected reference stations (red pentagram points) and IPPs (green points). In the processing, due to the lack of P1 observations at most stations, the CA code pseudoranges are used to extract the ionospheric delays by carrier-to-code leveling process. The satellites differential code biases (DCBs) are calibrated using the products processed by Wuhan University [45], and the receivers DCBs are estimated using the GIM products and the known satellites DCBs.

### 6.1. TEC maps

The ionospheric delay effect can be reflected by the values of TEC. Therefore, consecutive VTEC maps may allow detecting the local temporal-spatial variations in ionosphere. Based on the observations from CMONOC, China regional VTEC products are generated by applying the ionospheric TEC models mentioned above. The ionospheric activities during the campaign days have similar characteristics that the diurnal variations of the regional mean VTEC distribute in wave shapes with different maximum and minimum. The mean VTEC ranges from 10 to 80 TECU during campaign days. It reaches the maximum values of 55 to 80 TECU during the session from LT 12:00 to LT 14:00, and approaches the minimum values around 10 to 15 TECU in the local evening and before dawn.

**Figure 4:**
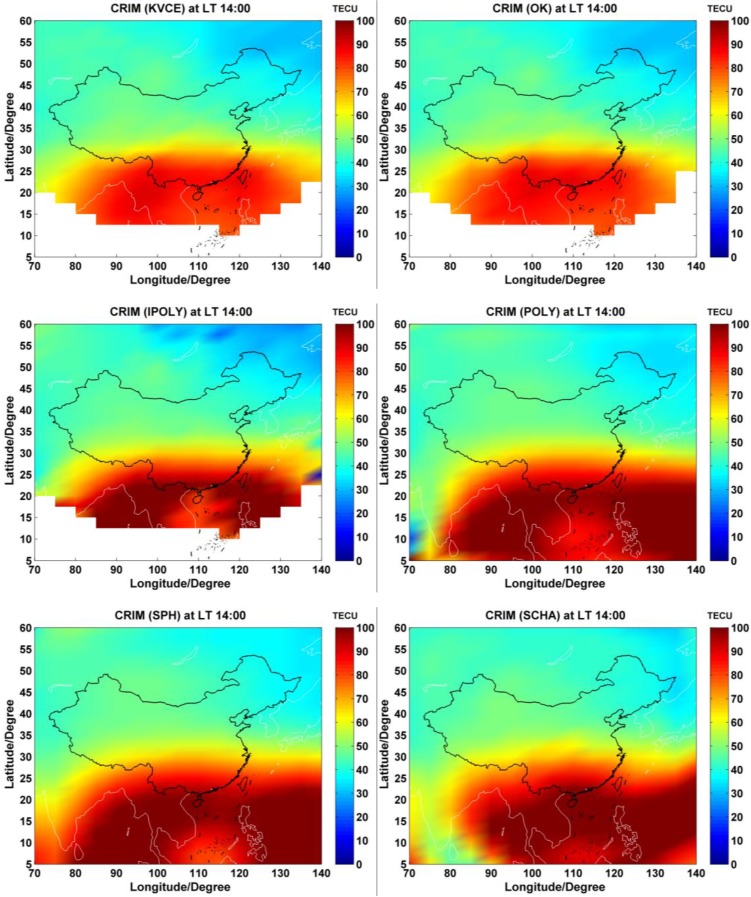
The VTEC maps obtained by different approaches at local time 14:00 on DOY 305 of 2014.

Figure 4 presents the examples of China regional instantaneous ionosphere VTEC maps by using the adopted models at the representative epoch with a high solar activity, LT 14:00, when the maximum ionospheric TEC appears commonly. The TEC value can reach nearly 100 TECU in low latitudes. In general, the phenomena that intense ionosphere variations and high values of ionospheric TEC occur frequently in low latitudes are attributed to the geomagnetic disturbances. While in middle latitudes, the ionospheric TEC varies smoothly, ranging from 30 to 50 TECU. Due to the lack of enough IPPs in the low latitude boundary areas, there are no interpolated estimations for KVCE, OK and IPOLY models. That is why there are blank areas in the first three maps of Figure 4. This situation restricts the correction effect of interpolation methods to some extent. Comparatively, as shown in the latter three maps of Figure 4, the function-based models, namely, POLY, SPH and SCHA, can provide the VTEC estimations for these areas, which are computed with their coefficients. But the map obtained from KVCE is similar to that of OK and seems to be a bit smoother compared with the other four approaches.

**Figure 5:**
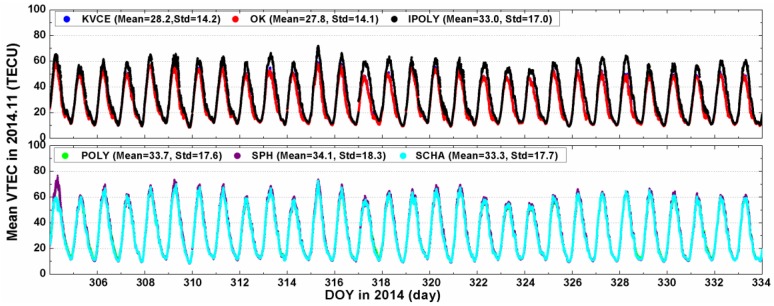
The mean values of VTEC for different approaches in 2014.11.

Figure 5 shows the time series of China regional mean VTEC values obtained from different models. In order to avoid overlapping of resultant curves, they are plotted into two groups to conveniently distinguish the differences among them. The distributions show similar ionospheric behaviors in spite of somewhat differences. The maximum VTEC, which can reach nearly 80 TECU, occurs during LT 12:00 to LT 14:00 due to intense activities of ionosphere. Then the VTEC gradually decreases to the minimum, ranging from 10 to 15 TECU. The overall mean and standard deviations of VTEC values for KVCE, OK, IPOLY, POLY, SPH and SCHA during the selected campaign days are equal to (28.2, 14.2) TECU, (27.8, 14.1) TECU, (33.0, 17.0) TECU, (33.7, 17.6) TECU, (34.1, 18.3) TECU and (33.3, 17.7) TECU, respectively. Since the mean VTEC values are different within 6 TECU, and the standard deviations are different by 4 TECU, we may conclude that all the methods under study achieve good agreements of results. Moreover, the results of the lowest mean value of OK may indicate that OK underestimates the ionospheric TEC a little more than KVCE, as compared to IPOLY, POLY, SPH and SCHA. Furthermore, the nearly same standard deviations of VTEC mean values of 14.2 TECU for KVCE and 14.1 TECU for OK indicate almost equivalent stabilities for both methods. The standard deviations of mean VTEC for other methods range from 17.0 to 18.3 TECU and are larger than those of KVCE and OK by about 3 TECU. Owing to the fact that KVCE neither underestimates nor overestimates the VTEC with high stability, this approach seems to be very promising for specifying the TEC over the investigated area.

### 6.2. Local Interpolation Residuals Analysis

The strategy of cross-validation is used to evaluate interpolation performances of different methods of KVCE, OK and IPOLY. For each IPP, its interpolated value of TEC is calculated in the same way as the procedure of IGP TEC estimation. This means that while polynomial is used for the interpolation, only those IPPs around the target IPP are used to calculate the polynomial coefficients. KVCE and OK are performed by following the same procedure. Taking ${\hat{I}}_{interp}$ to denote the STEC by interpolated VTEC conversion using the MSLM mapping function and *I* to denote the observed STEC at the same IPP, we can then compute Interpolation Root Mean Squared error (IRMS):

(46) $$\mathrm{IRMS}=\sqrt{\frac{\sum_{i=1}^{N}{\left({\hat{I}}_{interp}-I\right)}_{i}^{2}}{N}}$$

**Figure 6:**
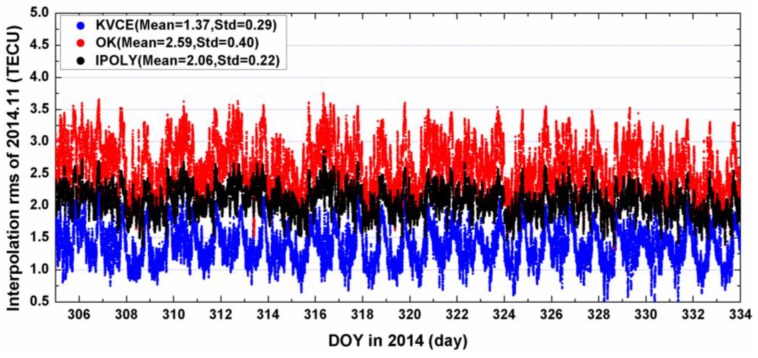
The IRMS of KVCE, OK and IPOLY in 2014.11.

**Figure 7:**
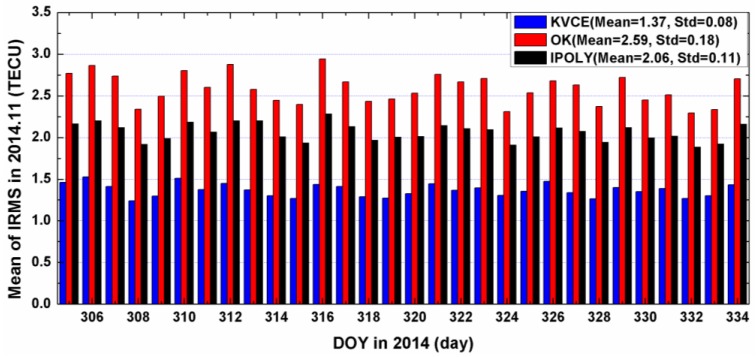
The daily mean of IRMS for KVCE, OK and IPOLY in 2014.11.

Figure 6 and Figure 7 present the IRMS results from KVCE, the ordinary Kriging and polynomial interpolation for DOY 305 to 334. The IRMS from the KVCE ranges from 0.5 to 2.5 TECU, while the IRMS values from the OK and IPOLY are from 1.5 to 3.5 TECU and 1.5 to 3.0 TECU, respectively. As shown in Figure 6, the standard deviations of the IMRS for KVCE, OK and IPOLY are 0.29 TECU, 0.40 TECU and 0.22 TECU, respectively. The comprehensive comparisons about the mean and standard deviation of IRMS indicate that KVCE is superior to OK and IPOLY. In addition, the overall statistical IRMS information can be seen obviously in Figure 7. The IRMS daily mean values of the three approaches are 1.37 TECU, 2.59 TECU and 2.06 TECU, respectively. The mean IRMS of KVCE is smaller than those of OK and IPOLY by about 1.2 TECU and 0.7 TECU, respectively. Besides, the standard deviation of the daily mean IRMS for KVCE is 0.08 TECU, achieving a better stability and/or precision than OK (0.18 TECU) and IPOLY (0.11 TECU). The polynomial interpolation fits the trend with the weights of the measurements, which is effective for estimating the deterministic part (trend) of TEC distribution in local areas; nevertheless, it does not use the random signals in spatial variations of TEC. Since OK uses a constant model as the trend, and since a trend model plays a dominating role in TEC modeling, this explains why the results of local IPOLY with a more flexible trend model are better than those of OK. On the other hand, KVCE not only gives full consideration to the balance between the weights of measurement noises and random signals but also the spatial relationships of the scattered IPP measurements. This probably explains why KVCE performance is superior over OK and IPOLY in local interpolation.

### 6.3. Regional Modeling Residuals Analysis

POLY, SPH and SCHA can be used to make regional TEC maps. The IPPs’ STEC can be computed with the fitted coefficients. Thus, we can also compute the residuals. To compare the performances of POLY, SPH and SCHA, we will use the following measure:

(47) $$MRMS=\sqrt{\frac{\sum_{i=1}^{N}{\left({\hat{I}}_{model}-I\right)}_{i}^{2}}{N}}$$

where the ${\hat{I}}_{model}$ is the STEC computed from the models above.

For KVCE, only IGP VTEC is estimated locally and VTEC grid models will be constructed here. With the estimates of the IGP VTEC, the IPPs with STEC surrounding IGP within a certain radius can be obtained sequentially. Then the KVCE residuals are computed. For the VTEC grid models from KVCE, the differences between the estimated IPP STEC and the observed IPP STEC are used to compute the modeling precision as follows:

(48) $$MRMS=\sqrt{\frac{\sum_{i=1}^{N_{IGP}}{\left(\sum_{j=1}^{M_{i}}{\left({\hat{I}}_{KVCE}-I\right)}_{j}^{2}\right)}_{i}}{\sum_{i=1}^{N}M_{i}}}$$

where the ${\hat{I}}_{KVCE}$ is the *j-*th estimated IPP STEC, $M_{i}$ is the number of IPPs surrounding the *i-*th IGP, $N_{IGP}$ is the number of IGPs.

**Figure 8:**
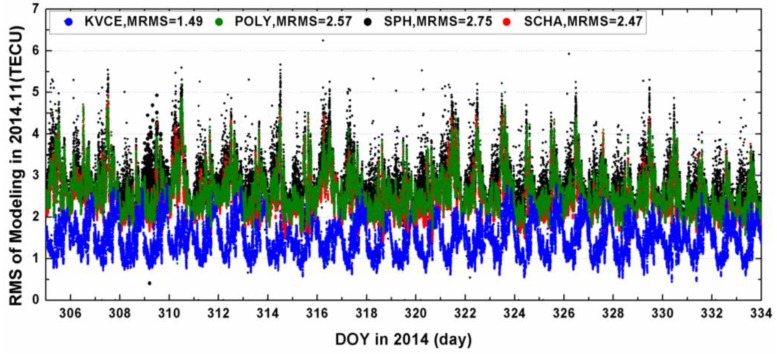
The MRMS of KVCE and TEC modelings with polynomial functions, spherical harmonic functions and spherical cap harmonic functions.

Figure 8 plots the mean Modeling RMS (MRMS) with the results from KVCE, POLY, SCHA and SPH. The MRMS from KVCE ranges from 0.5 to 3 TECU, with the mean value of 1.49 TECU, while the MRMS values from POLY, SPH and SCHA are about 2.57 TECU, 2.75 TECU and 2.47 TECU, respectively. Obviously, the modeling fitting of KVCE is better than those of SCHA, POLY and SPH methods. This is reasonable because KVCE grid models take local fitting around IGPs while POLY, SCHA and SPH make regional fitting over China. Even if POLY is used both for local interpolation for IGPs with local IPPs around it and for regional fitting over China area, the fitting residuals are at a similar level of around from 2 to 4 TECU (compare Figure 8 and Figure 6). SCHA and SPH have a similar residual level as POLY in the regional fitting over China.

### 6.4. TEC RMS Maps

After estimating the ionospheric delays at IGPs, we can further compute the estimation accuracy, namely, RMS or grid sigma σ. The estimation accuracy of KVCE, POLY, SCHA and SPH can be computed by applying the variance-covariance propagation law expressed by Equation (37). The estimation accuracy of OK can be obtained by Equation (12). With the data from this CMONOC network, the daily mean RMS values of the POLY, SCHA and SPH are equal to 10.5 TECU, 9.05 TECU and 9.44 TECU, respectively. These numbers are significantly larger than those of KVCE and OK. Therefore, we focus on the comparison of results between KVCE and OK in this section.

**Figure 9:**
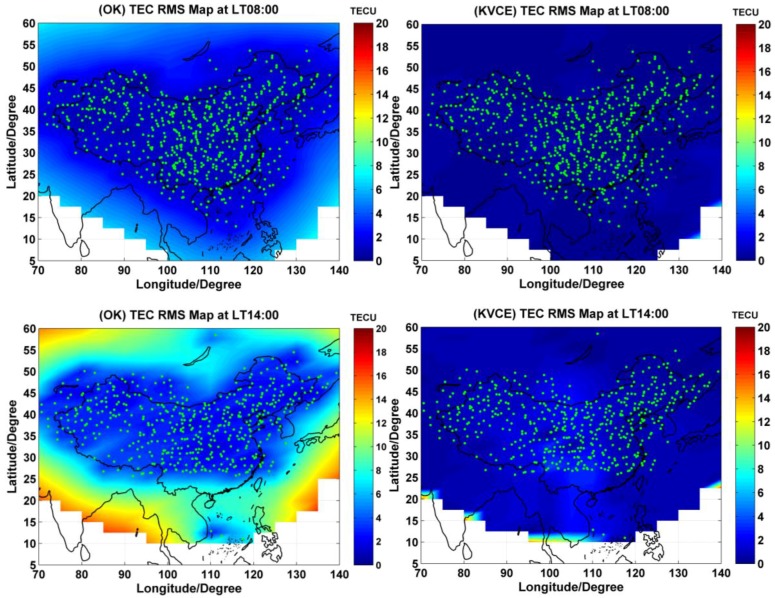

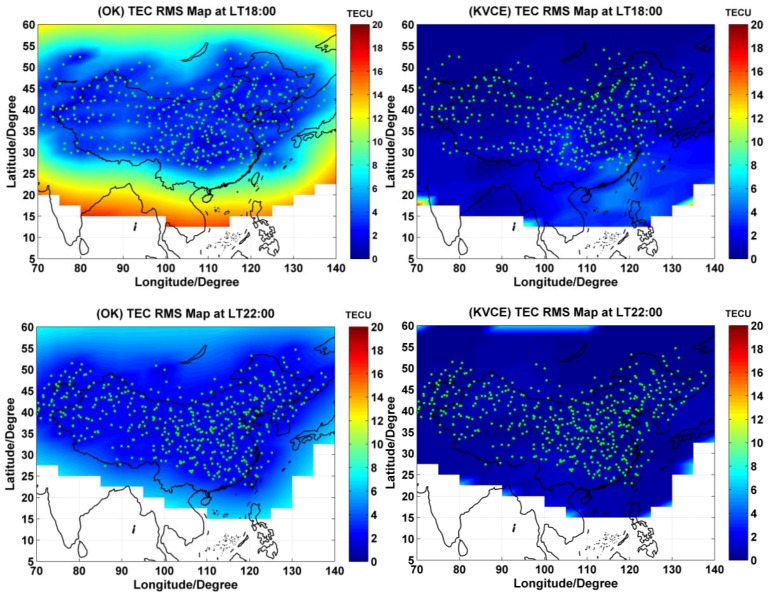
The grid sigma estimated by OK at different local time (left column) and KVCE (right column) on DOY 305.

Figure 9 presents the nephograms of estimation accuracy over China at four different local time epochs, morning (LT 08:00), noon (LT 14:00), afternoon (LT 18:00) and evening (LT 22:00) on DOY 305, with the subplots in the left and right columns of Figure 9 showing the IGPs RMS results of OK and KVCE, respectively. Due to the lack of IPPs at the boundary, the IGPs in low latitudes have no interpolation values in the blank area shown in Figure 9, which will be called as invalid grids in the following.

**Figure 10:**
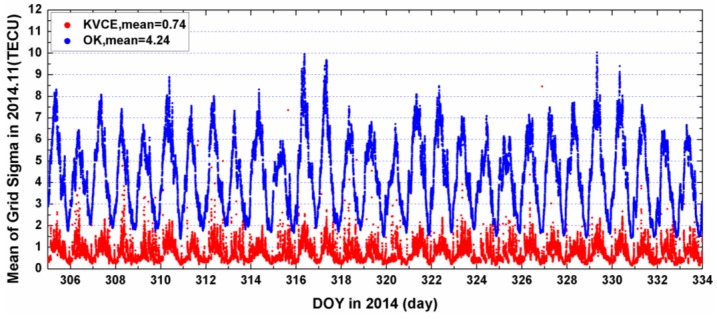
The mean grid sigma of KVCE (red dots) and OK (blue dots) in 2014.11.

**Figure 11:**
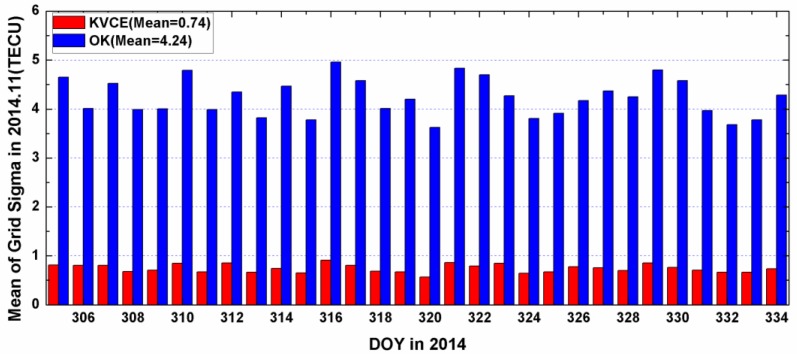
The daily mean grid sigma of KVCE and OK in 2014.11.

The accuracy of the estimation results depends on the quantity of the measurements and the degree of ionospheric activity. In general, the accuracy in the central region is higher than that along the boundary. For the subplots in the left column of Figure 9, the OK RMS at LT 08:00 and LT 22:00 is from 0 to 8 TECU, and the characteristics of IGPs RMS distribution are inconspicuous for the quiet ionospheric activities, when compared with the IPPs distribution. At LT 14:00 and LT 18:00, the RMS is within 2 to 20 TECU, and the magnitude of IGPs RMS depends on the density of the IPPs. The pattern of these maps is apparently the same as that of the IPPs distributions. This result indicates that the RMS at noon and in the afternoon is larger than that in the morning and evening, and has evident variation along with the distribution of IPPs. The major reason for this phenomenon is the disturbed ionosphere condition in the daytime, especially at noon and in the afternoon. The KVCE RMS results, as shown in the right column of Figure 9, are within 6 TECU, which are better than those of OK interpolation. The abnormal phenomena appear at LT 14:00 in the KVCE ionosphere map, which shows that the central area has a larger RMS than the boundary areas. A possible reason is likely due to the non-convergence of the VCE estimates. The accuracy obtained in the morning and at night is better than that at noon and in the afternoon. In general, KVCE is better than OK, no matter whether the points are in the boundary area. Moreover, occurrence of the boundary errors have a big impact on the results which are presented in the Figure 9, as well as the following Figure 10 and Figure 11. Certainly, if the points in the boundary area are not taken into account, the differences between Kriging and KVCE will be smaller. Nevertheless, we believe that to fairly compare both methods, it is more reasonable to count on all the points. In other words, the advantage of KVCE becomes even clearer in the boundary area.

**Figure 12:**
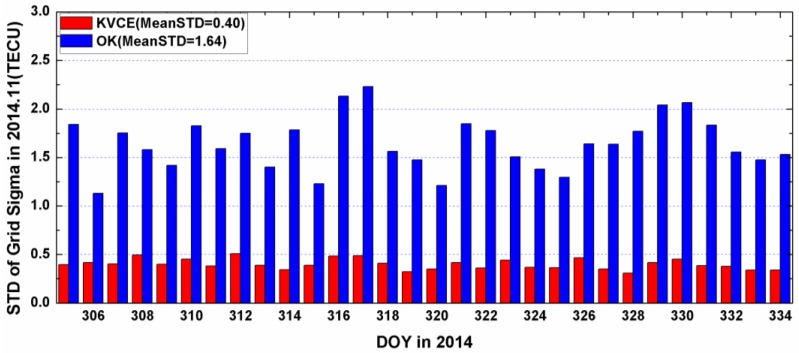
The STD of grid sigma of KVCE and OK in 2014.11.

**Figure 13:**
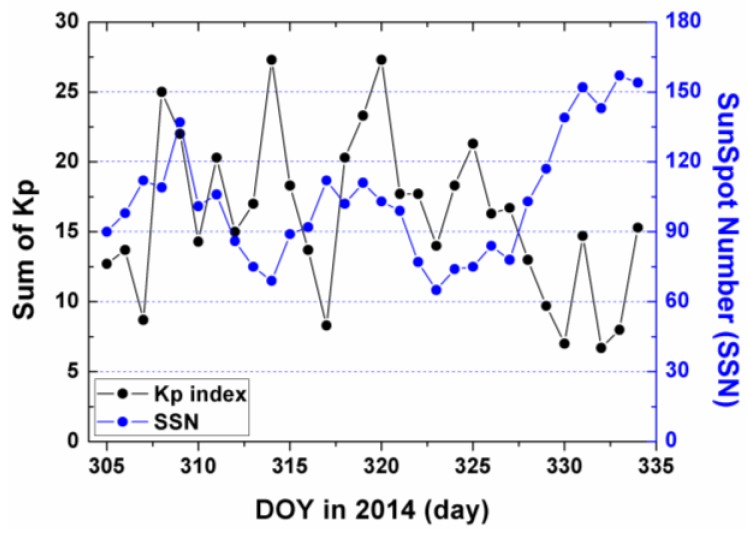
The Kp Index and Sunspot Number in 2014.11.

Figure 10 and Figure 11 plot the distribution of time series for the epoch mean RMS and daily mean RMS of both methods. Although the patterns of the mean RMS of KVCE and OK are similar, KVCE is clearly significantly better than OK, as can also be further confirmed from the standard deviation statistics plotted in Figure 12. The daily mean RMS of KVCE is less than 1 TECU and that of OK is about 4 TECU. The former is about four times better than the latter in terms of RMS. The abnormal phenomena appear at LT 14:00 in the KVCE ionosphere map, which shows that the central area has a larger RMS than the boundary areas. The possible reason is likely due to the non-convergence of the VCE estimates. Furthermore, the accuracy estimated in the morning and at night is better than that at noon and in the afternoon. Figure 13 also presents the Kp index and SunSpot Number (SSN) showing that the daily sum Kp index and SSN are lower than 30 and 180, respectively. Generally, the ionospheric activity is relatively stable for the campaign days. It is nice confirmation of the KVCE method which gives much more reliable estimations as shown in Figure 12.

## 7. Conclusions

We have proposed a Kriging method with unknown variance components to interpolate ionospheric delays for use in real-time, which is an extension of the works by Xu et al. [35] and Xu [39]. As a result, we are able to correctly determine the weighting factors of measurement noises and random signals. The proposed new method has been applied to GPS data collected in 2014 from the Crustal Movement Observation Network of China (CMONOC) and compared with other methods such as ordinary Kriging, polynomial interpolation, low-degree spherical harmonic function models, polynomial function models and spherical cap harmonic analysis models in terms of TEC maps comparisons, local interpolation, regional modeling and VTEC accuracy at ionospheric grid points.

The results show that the ionospheric vertical structures obtained by applying these methods reach a good agreement, with the daily mean VTEC variations ranging from 10 to 80 TECU and the overall mean values from 27.8 to 34.1 TECU during the campaign days. The mean VTEC value from KVCE is close to that from OK, which is lower by about 6 TECU than IPOLY, POLY, SPH and SCHA but with a better stability of around 3 TECU. The VTEC maps derived from KVCE and OK are smoother than those of other four methods, but OK tends to underestimate VTEC a little more than KVCE. This fact indicates that KVCE seems to be more promising for reconstructing the TEC over China regional area. In addition, the local interpolation precision of our proposed method is 0.5–2.5 TECU with a mean value of 1.37 TECU, which is smaller than that of the ordinary Kriging and polynomial interpolation by about 1.2 TECU and 0.7 TECU, respectively. The regional modeling precision from KVCE ranges from 0.5 to 3 TECU with an overall mean value of 1.5 TECU, which is smaller than those from the function-based models by about 1 TECU. The estimation accuracy at ionospheric grid points from our new method remains within 6 TECU, with a daily mean of 0.74 TECU and a standard deviation of 0.4 TECU. The results are better than those from the ordinary Kriging, which can even reach 20 TECU under intense solar activities. In addition, the advantage of KVCE becomes even clearer than OK in the boundary area when all the points are counted. The comprehensive analysis results in terms of TEC maps, interpolation, modelling and estimation accuracy with ionospheric grid points have clearly shown that the proposed Kriging method with variance components has the best performance and can produce more rational, and accurate ionospheric TEC than all the other methods used for comparison in this paper.
